# Supplementary material for: MetaRibo-Seq measures translation in microbiomes
Source: Nat Commun. 2020 Jun 29;11:3268. doi: 10.1038/s41467-020-17081-z (PMC7324362; doi:10.1038/s41467-020-17081-z)
Supplement: Supplementary file 10 — Supplementary Data 7 [file 41467_2020_17081_MOESM10_ESM.zip › File2/Confidence_High_Taxonomy/367892_out.krona.html]

Javascript must be enabled to view this page.

members
magnitude
magnitudeUnassigned
count
unassigned
taxon
rank

367892\_out

50

2
superkingdom
50

1239
50
phylum

186801
class
50

6
50

SRS011405\_contig\_number\_5458SRS014979\_contig\_number\_21300SRS049959\_contig\_number\_22564SRS063370\_contig\_number\_10688SRS063518\_contig\_number\_12870SRS147919\_contig\_number\_15496
186802
order

541000
2
family

216851
1
genus

species
1

SRS147377\_contig\_number\_300
853

species
1

SRS097889\_contig\_number\_28914
39492

186803
family
8

841
4
genus

1
species

SRS013951\_contig\_number\_contig-100\_5.219507
2293144


SRS015663\_contig\_number\_24131
2293129
species
1

species
2
166486

SRS019267\_contig\_number\_9244SRS098827\_contig\_number\_13369

28050
genus
4

species
4

SRS043667\_contig\_number\_5609SRS050925\_contig\_number\_22690SRS143070\_contig\_number\_15401SRS144537\_contig\_number\_24119
28052

family
34
186806

1730
genus
34

18
species
142586

SRS013476\_contig\_number\_37735SRS014235\_contig\_number\_38767SRS015431\_contig\_number\_8414SRS017191\_contig\_number\_contig-100\_921.111547SRS047044\_contig\_number\_18399SRS051031\_contig\_number\_12047SRS052027\_contig\_number\_4088SRS056519\_contig\_number\_contig-100\_177.110399SRS063985\_contig\_number\_26830SRS077502\_contig\_number\_1016SRS078176\_contig\_number\_13655SRS097920\_contig\_number\_11297SRS143342\_contig\_number\_2554SRS143780\_contig\_number\_32865SRS144135\_contig\_number\_34667SRS146812\_contig\_number\_60387SRS148091\_contig\_number\_9735SRS149181\_contig\_number\_11857

species
1
39485

SRS058723\_contig\_number\_3328

13
species
1262892

SRS014855\_contig\_number\_11220SRS018984\_contig\_number\_30715SRS023346\_contig\_number\_5211SRS043701\_contig\_number\_10983SRS063040\_contig\_number\_32256SRS065504\_contig\_number\_contig-100\_8.210483SRS077335\_contig\_number\_1654SRS100021\_contig\_number\_10884SRS104311\_contig\_number\_38165SRS142890\_contig\_number\_19579SRS144183\_contig\_number\_39574SRS147271\_contig\_number\_789SRS148253\_contig\_number\_8052


SRS015190\_contig\_number\_2641SRS1055022\_contig\_number\_20165
1897026
2
species
